# Quality of Life, Symptom Burden, and Associated Factors Among Patients With Lung Cancer in Sub-Saharan Africa: Cross-Sectional Study

**DOI:** 10.2196/87232

**Published:** 2026-07-16

**Authors:** Mpho Ratshikana, Kija Malale, Abdul-Rauf Sayed, Oluwatosin Ayeni, Peedi Mathobela, Rofhiwa Mathiba, Charmaine Blanchard, Merika Tsitsi, Daniel Osei-Fofie, Anita Graham, Herry Owuor Dhudha, Nestory Masalu

**Affiliations:** 1 Department of Internal Medicine Chris Hani Baragwanath Academic Hospital Johannesburg, Gauteng South Africa; 2 Soweto Comprehensive Cancer Center Chris Hani Baragwanath Hospital Johannesburg, Gauteng South Africa; 3 Department of Internal Medicine Faculty of Health Sciences University of Witwatersrand Johannesburg, Gauteng South Africa; 4 Department of Clinical Nursing Catholic University of Health and Allied Sciences Mwanza United Republic of Tanzania; 5 Department of Oncology Bugando Medical Centre Mwanza United Republic of Tanzania; 6 Bristol-Myers Squibb Foundation (BMSF) Johannesburg, Gauteng South Africa; 7 Department of Radiation Oncology Faculty of Health Sciences University of the Witwatersrand Johannesburg, Gauteng South Africa; 8 Strengthening Oncology Services Research Unit Faculty of Health Sciences University of the Witwatersrand Johannesburg, Gauteng South Africa; 9 Department of Oncology Robert Mangaliso Sobukwe Hospital Kimberly, Northern Cape South Africa; 10 Division of Medical Oncology University of Witwatersrand Faculty of Health Sciences Johannesburg, Gauteng South Africa; 11 Department of Oncology Catholic University of Health and Allied Sciences Mwanza United Republic of Tanzania

**Keywords:** cancer, lung, quality of life, QoL, symptoms

## Abstract

**Background:**

Lung cancer remains a major contributor to cancer mortality in sub-Saharan Africa (SSA), where late diagnosis, driven by low awareness, sociocultural barriers, and health system constraints, limits effective treatment. Despite the growing burden, evidence on patients’ quality of life (QoL) and symptom experience in SSA is limited.

**Objective:**

This study aimed to describe the common symptoms and QoL of patients with lung cancer treated at 2 hospitals in SSA, and to investigate the association of demographics, clinical characteristics, and symptom burden with QoL.

**Methods:**

This was a cross-sectional study that consecutively recruited patients with lung cancer from 2 teaching hospitals in SSA: Bugando Medical Centre (BMC) in Tanzania and the University of the Witwatersrand Centre of Respiratory Excellence (WITS-CORE) in South Africa. Data collected included demographics, clinical information, and performance status using the Eastern Cooperative Oncological Group Performance Scale (ECOG-PS). Health-related QoL was assessed using the 30-item European Organization for Research and Treatment of Cancer Quality of Life Questionnaire Core 30 (EORTC QLQ-C30). The study followed all ethical procedures, and data were analyzed using both descriptive and inferential statistics in Stata 18. A *P* value of <.05 was considered statistically significant.

**Results:**

A total of 174 patients with lung cancer were enrolled across the 2 sites. The score on the EORTC QLQ-C30 global health status/QoL subscale was low, with a median of 41.67 (IQR 33.33-41.67), and it varied by site. Patients from WITS-CORE demonstrated higher social functioning scores, while those from BMC reported greater financial difficulties. A low global health status/QoL score was independently associated with the BMC site (adjusted odds ratio [aOR] 3.5, 95% CI 1.3-9.3) and poor performance status (ECOG-PS 3-4; aOR 3.4, 95% CI 1.2-6.6). Furthermore, symptoms such as nausea and vomiting, pain, dyspnea, insomnia, appetite loss, diarrhea, and financial struggles were all associated with a low global health status/QoL score.

**Conclusions:**

QoL among patients with lung cancer in SSA is poor. Low QoL is strongly associated with the Multinational Lung Cancer Control Program study site, poor performance status, and a range of symptoms and financial difficulties. Addressing these factors may help to improve patient outcomes and well-being in SSA.

## Introduction

GLOBOCAN estimates indicated that more than 19 million new cancer cases occurred worldwide in 2022, with lung cancer accounting for 12.4%, making it the most diagnosed cancer [1]. Although the incidence of lung cancer in Africa is lower than in high-income countries, it remains among the top 5 cancers in both sexes, and it is the fourth leading cause of cancer-related mortality, with southern Africa bearing the highest burden on the continent [1]. In sub-Saharan Africa (SSA), most patients present at advanced stages, partly due to limited community awareness, alongside the influence of sociocultural factors, health system constraints, and gaps in health care provider knowledge [2-5]. These challenges contribute to delayed diagnosis, limited treatment options, and consequently high mortality rates [6].

Lung cancer is associated with a substantial symptom burden, including fatigue, dyspnea, cough, pain, appetite loss, and hemoptysis. However, in SSA, late presentation often results in more severe complications, such as brain metastases, pleural effusion, spinal cord compression, and superior vena cava obstruction syndrome [7,8]. As a result, treatment is frequently limited to palliative approaches, including chemotherapy and radiotherapy, aimed primarily at improving quality of life (QoL).

QoL is a critical outcome in cancer care, particularly for patients with advanced disease [9-11]. Lung cancer significantly compromises physical, emotional, social, and spiritual well-being, with strong evidence linking QoL to symptom burden, clinical characteristics, and sociodemographic factors [12,13]. Importantly, better QoL is associated with improved health outcomes and survival [9,14]. Interventions such as chemotherapy, targeted therapy, immunotherapy, radiotherapy, and palliative care have demonstrated benefits in symptom control, functional status, and overall well-being [10,15]. However, most evidence on symptom burden and QoL originates from high-income settings, with limited data from SSA.

This study aimed to describe the common symptoms and QoL of patients with lung cancer receiving care at 2 tertiary institutions in SSA: the University of the Witwatersrand (WITS) Centre for Respiratory Excellence (CORE) in South Africa and Bugando Medical Centre (BMC) in Tanzania. The study also examined the association between sociodemographic and clinical characteristics, symptom burden, and QoL. We hypothesized that patients with lung cancer in SSA would experience a high symptom burden and poor QoL, with significant associations between these outcomes and patient-related factors.

## Methods

### Ethical Considerations

This study was conducted in accordance with the ethical principles outlined in the Declaration of Helsinki, including respect for individuals, their right to make informed decisions, and the protection of their health and rights. Ethics approval was obtained from the Human Research Ethics Committee (Medical), WITS (M180436). In Tanzania, the ethics approval was obtained from the Joint Catholic University of Health and Allied Sciences and BMC Research Ethics and Review Committee (CREC/278/2018) and the National Institute for Medical Research (MR/53/100/598). Informed consent was obtained from all participants before participating in this study. Participants’ privacy and confidentiality were strictly maintained throughout the study by anonymizing data and securely handling all research information.

### Setting

The study was conducted at 2 teaching hospitals participating in the Multinational Lung Cancer Control Program (MLCCP) in 2 SSA countries: Tanzania and South Africa. WITS-CORE is a specialized respiratory unit providing comprehensive lung cancer services to patients in Johannesburg, South Africa. It is based at Helen Joseph Hospital, a teaching hospital affiliated with the WITS. BMC is a consultant-teaching and referral hospital for the Lake and Western zone of the United Republic of Tanzania affiliated with the Catholic University of Health and Allied Sciences in Mwanza, Tanzania. The hospital’s Oncology department is the only cancer center in the Lake and Western zone of United Republic of Tanzania that provides comprehensive cancer care with outreach services to urban and rural areas.

### Design and Participants

This cross-sectional study consecutively recruited patients with lung cancer from 2 teaching hospitals in SSA. The inclusion criteria were patients aged 18 years or older, a confirmed primary lung cancer diagnosis, and being physically and mentally able to participate in the study. A similar protocol was applied across the study sites; once the diagnosis was confirmed and before treatment began, patients were invited to participate in the study. Those who consented were enrolled, and sociodemographic and clinical information were collected. Thereafter, the questionnaire was administered in English (WITS-CORE) or Swahili (BMC) and, where needed, translated into the vernacular. Data collection was conducted for 1 year, from August 2020 to August 2021.

### Study Measures

Trained nurses and interviewers conducted interviews following the standardized protocol to obtain demographic information (age, gender, marital status, education level, employment status, smoking, and mining history) and clinical information (histology, clinical staging, Eastern Cooperative Oncological Group Performance Scale [ECOG-PS], weight loss, active or current tuberculosis, and other comorbidities). The ECOG-PS was used to determine the performance status of patients [16].

QoL, functional status, symptom burden, and financial impact were assessed using the 30-item European Organization for Research and Treatment of Cancer Quality of Life Questionnaire Core 30 (EORTC QLQ-C30) [17]. The EORTC QLQ-C30 is composed of 3 multi-item or single-item subscales: the global health status/QoL scale, 5 functional scales (physical, role, social, emotional, and cognitive functioning), 8 symptom scales (fatigue, pain, nausea and vomiting, dyspnea, insomnia, appetite loss, constipation, and diarrhea), and a financial impact scale. Each item has four possible response alternatives: (1) “not at all,” (2) “a little,” (3) “quite a bit,” and (4) “very much.” The responses to the scale items refer to “last week,” with the exception of the patient’s physical performance scale, where the timeframe is the present. The scores of each scale were calculated in accordance with the EORTC QLQ-C 30 scoring manual [11]. The sum of items in each category was added, and the total was divided by the number of questions in the category. A linear transformation was then undertaken to convert the score to a percentage scale. All the scales and single-item measures range from 0 to 100. Higher scores on the functional and QoL scales translated to better health-related QoL, whereas higher scores on the symptom scales translated to a higher level of symptoms or problems.

### Data Analysis

Study data were collected and managed using REDCap tools hosted at WITS. Patients’ demographics, clinical characteristics, and health-related QoL information were extracted from the REDCap database and exported to Stata (version 18; StataCorp) for statistical analysis [18,19]. Categorical variables were presented as frequency tables, and continuous variables were presented as descriptive measures, expressed as medians and interquartile ranges (IQRs). The nonparametric Wilcoxon rank-sum test was used to compare the QoL scores between study sites. Univariate and multivariate binary logistic regression analysis were performed to evaluate the association between QoL and sociodemographic factors, clinical characteristics, and symptom burden. Consistent with previous studies, QoL was dichotomized using the median (50th percentile) as the cut-off point: a score greater than or equal to 50 was defined as “above-average QoL,” while a score less than 50 was defined as “below-average QoL” [20]. Odds ratios (ORs) were used to test the association between variables, and 95% CI that did not span unity were considered as thresholds of statistical significance. Adjusted ORs (aORs) were used in multivariate analysis.

## Results

### Sociodemographic Characteristics

A total of 174 patients with lung cancer consented and were enrolled across the 2 sites: 35 (20.1%) at BMC, and 139 (79.9%) at WITS-CORE. Table 1 shows the sociodemographic characteristics of the participants. Most patients were male (n=115, 66.1%), unemployed or retired (n=135, 80%), with a mean age of 61.6 (SD 11.6) years. There were significant differences in the demographic characteristics between the patients at the 2 MLCCP sites. More WITS-CORE patients were male (97/139, 69.8%) than those at BMC (18/35, 51.4%; *P*=.04). More than two-thirds of the patients with lung cancer at WITS-CORE (98/139, 70.5%) had a high school or higher level of education compared with participants at BMC (8/35, 22.9%; *P*<.001). A significant proportion of WITS-CORE patients (108/139, 77.7%) were current or ex-smokers compared with patients at BMC (8/35, 22.9%; *P*<.001).

**Table 1:** Sociodemographic characteristics of the participants.

Sociodemographic variables		BMC^a^ (n=35)	WITS-CORE^b^ (n=139)	Total (n=174)	*P* value
**Age group (y), n (%)**					
	<40	4 (11.4)	5 (3.6)	9 (5.2)	—^c^
	40-49	4 (11.4)	10 (7.2)	14 (8)	—
	50-59	10 (28.6)	32 (23)	42 (24.1)	—
	60-69	11 (31.4)	56 (40.3)	67 (38.5)	—
	≥70	6 (17.1)	36 (25.9)	42 (24.1)	—
Age (y), mean (SD)		58.0 (13.4)	62.5 (12.0)	61.6 (11.6)	.04
**Sex, n (%)**					
	Female	17 (48.6)	42 (30.2)	59 (33.9)	—
	Male	18 (51.4)	97 (69.8)	115 (66.1)	.04
**Educational level, n (%)**					
	≤Primary education	27 (77.1)	41 (29.5)	68 (39.1)	—
	≥High school	8 (22.9)	98 (70.5)	106 (60.9)	<.001
**Marital status, n (%)**					
	Married or in partnership	29 (82.9)	79 (58.5)	108 (62.1)	—
	Single, divorced, or widowed	6 (17.1)	60 (41.5)	66 (37.9)	<.001
**Occupation, n (%)**					
	Employed	10 (28.6)	29 (20.9)	39 (20)	—
	Unemployed or retired	25 (71.4)	110 (79.1)	135 (80)	.33
**Smoking, n (%)**					
	Never smoked	27 (77.1)	31 (22.3)	58 (33.3)	—
	Current or ex-smoker	8 (22.9)	108 (77.7)	116 (66.7)	<.001
**Ever a miner, n (%)**					
	Yes	0 (0.0)	19 (13.7)	19 (10.9)	—
	No	35 (100)	120 (86.3)	155 (89.1)	.02

^a^BMC: Bugando Medical Centre.

^b^WITS-CORE: University of the Witwatersrand Center of Respiratory Excellence.

^c^Not applicable.

### Clinical Characteristics

Most patients (157/174, 90.2%) were diagnosed with non–small cell lung cancer (NSCLC). Most presented with late-stage (III-IV) diseases, including 96.7% (148/153) of the patients with NSCLC and 85.7% (18/21) of those with small cell lung cancer. Only 4% (5/126) of the WITS-CORE patients with NSCLC presented with early stage (I-II) disease (Table 2). Most patients at BMC (30/35, 85.7%) presented with weight loss compared with patients at WITS-CORE (94/139, 67.6%; *P*=.04). Similarly, a significant proportion of BMC patients had current tuberculosis (12/35, 34.3%) compared with those at WITS-CORE (5/139, 3.6%; *P*<.001). A higher proportion of WITS-CORE patients presented with one or more comorbidities (81/139, 58.3%) compared with patients at BMC (14/35, 40%; *P*<.001).

**Table 2:** Clinical characteristics of participants.

Clinical variables			BMC^a^ (n=35), n (%)		WITS-CORE^b^ (n=139), n (%)		Total (n=174), n (%)		*P* value
**Clinical staging**									
	NSCLC^c^ stage I-II^d^	0 (0)		5 (4)		5 (3.3)		—^e^	
	NSCLC stage III-IV	27 (100)		121 (96)		148 (96.7)		.29	
	SCLC^f^ (limited)^g^	0 (0.0)		3 (23.1)		3 (14.3)		—	
	SCLC (extensive)	8 (100)		10 (76.9)		18 (85.7)		.14	
**ECOG^h^** **performance status**									
	0-2	23 (65.7)		100 (72.7)		123 (70.7)		—	
	3-4	12 (34.3)		39 (27.3)		51 (29.3)		.47	
**Weight loss**									
	No	5 (14.3)		45 (32.4)		50 (28.7)		—	
	Yes	30 (85.7)		94 (67.6)		124 (71.3)		.04	
**Current tuberculosis**									
	No	23 (65.7)		134 (96.4)		157 (90.2)		—	
	Yes	12 (34.3)		5 (3.6)		17 (9.8)		<.001	
**HIV status**									
	HIV negative	35 (100)		117 (84.2)		152 (87.4)		—	
	HIV positive	0 (0)		22 (15.8)		22 (12.6)		.01	
**Comorbidity**									
	No	21 (60)		58 (41.7)		79 (45.4)		—	
	Yes	14 (40)		81 (58.3)		95 (54.6)		.05	

^a^BMC: Bugando Medical Centre.

^b^WITS-CORE: University of the Witwatersrand Center of Respiratory Excellence.

^c^NSCLC: non–small cell lung cancer.

^d^For NSCLC staging, denominators were 27 (BMC), 126 (WITS-CORE), and 153 (total).

^e^Not applicable.

^f^SCLC: small cell lung cancer.

^g^For SCLC staging, denominators were 8 (BMC), 13 (WITS-CORE), and 21 (total).

^h^ECOG: Eastern Cooperative Oncological Group.

### QoL, Functional Status, and Symptom Burden

Overall, the EORTC QLQ-C30 global health status/QoL subscale score was low (Table 3); the median score was 41.7 (IQR 33.33-41.67). WITS-CORE patients had higher QoL (*P*=.004) and social functioning scores (*P*<.001) compared with patients at BMC. Financial difficulty was significantly higher among BMC patients compared with WITS-CORE (*P*<.001). Overall, the highest symptom scores reported were pain (66.67, IQR 33.33-66.67) and fatigue (55.56, IQR 44.44-55.56). Pain and insomnia scores were significantly higher among patients at BMC, with median scores of 83.33 (IQR 50.00-83.33; *P*<.001) and 66.67 (IQR 33.33-66.67; *P*<.001), respectively, compared with patients at WITS-CORE.

**Table 3:** Quality of life, functional status, and symptom burden of patients with lung cancer at 2 teaching hospitals across sub-Saharan Africa.

Assessment aspect			Total (n=174), median (IQR)		BMC^a^ (n=35), median (IQR)		WITS-CORE^b^ (n=139), median (IQR)		*P* value^c^
Global health status/quality of life			41.67 (33.33-41.67)		33.33 (16.67-33.33)		41.67 (33.33-41.67)		.004
**Functional scales**									
	Physical functioning	46.67 (26.67-46.67)		66.67 (6.67-66.67)		46.67 (33.33-46.67)		.58	
	Role functioning	33.33 (16.67-33.33)		50.00 (0.00-50.00)		33.33 (16.67-33.33)		.45	
	Emotional functioning	66.67 (50.00-66.67)		75.00 (41.67-75.00)		66.67 (50.00-66.67)		.76	
	Cognitive functioning	83.33 (50.00-83.33)		83.33 (50.00-83.33)		83.33 (50.00-83.33)		.30	
	Social functioning	33.33 (0.00-33.33)		0.00 (0.00-0.00)		50.00 (33.33-50.00)		<.001	
**Symptoms scale**									
	Fatigue	55.56 (44.44-55.56)		55.56 (33.33-55.56)		55.56 (44.44-55.56)		.80	
	Nausea and vomiting	0.00 (0.00-0.00)		16.67 (0.00-16.67)		0.00 (0.00-0.00)		<.001	
	Pain	66.67 (33.33-66.67)		83.33 (50.00-83.33)		50.00 (33.33-50.00)		<.001	
	Dyspnea	33.33 (33.33-33.33)		33.33 (0.00-33.33)		33.33 (33.33-33.33)		.36	
	Insomnia	33.33 (0.00-33.33)		66.67 (33.33-66.67)		33.33 (0.00-33.33)		<.001	
	Appetite loss	33.33 (0.00-33.33)		33.33 (0.00-33.33)		33.33 (0.00-33.33)		.09	
	Constipation	0.00 (0.00-0.00)		0.00 (0.00-0.00)		0.00 (0.00-0.00)		<.001^d^	
	Diarrhea	0.00 (0.00-0.00)		0.00 (0.00-0.00)		0.00 (0.00-0.00)		.01^d^	
**Financial impact scale**									
	Financial difficulties	66.67 (33.33-66.67)		100.00 (100.00-100.00)		66.67 (33.33-66.67)		<.001	

^a^BMC: Bugando Medical Centre.

^b^WITS-CORE: University of the Witwatersrand Centre of Respiratory Excellence.

^c^Significant at *P*<.003 (Bonferroni corrected).

^d^Despite identical medians and IQRs in both groups for constipation and diarrhea, the statistical test revealed a significant difference (*P*<.001) due to the presence of small but systematic differences in the distribution of values that are not captured by median or IQR.

### Associations Between Sociodemographics, Clinical Characteristics, and QoL

The results of the multiple logistic regression analysis using the dependent variable (QoL) as a binary outcome based on the 50th percentile or median (median QoL score <50 and median QoL score ≥50) are shown in Table 4. A significantly higher proportion of patients with lung cancer at BMC had below-average QoL compared with patients at WITS-CORE (aOR 3.5, 95% CI 1.3-9.3). Poor ECOG-PS score (3-4) was associated with poorer QoL score (aOR 3.4, 95% CI 1.2-6.6).

**Table 4:** Associations between sociodemographics, clinical characteristics, and quality of life (QoL) of patients with lung cancer at 2 teaching hospitals across sub-Saharan Africa.

Variables			Below-average QoL (<50; n=97), n (%)		Above-average QoL (≥50; n=77), n (%)		OR^a^ (95% CI)		*P* value		aOR^b^ (95% CI)^c^
**Age group (y)**											
	<60	38 (39.2)		27 (35.1)		Reference		—^d^		Reference	
	≥60	59 (60.8)		50 (64.9)		1.1 (0.6-1.9)		.84		1.1 (0.5-2.1)	
**Sex**											
	Male	64 (66)		51 (66.2)		Reference		—		Reference	
	Female	33 (34)		26 (33.8)		1.1 (0.6-1.9)		.99		1.0 (0.5-2.0)	
**Education**											
	≤Primary education	45 (46.4)		23 (29.9)		1.8 (0.9-3.5)		.08		1.8 (1.0-3.1)	
	≥Secondary education	52 (53.6)		54 (70.1)		Reference		—		Reference	
**Occupation**											
	Employed	18 (18.6)		21 (27.3)		Reference		—		Reference	
	Unemployed or retired	79 (81.4)		56 (72.7)		1.4 (0.7-2.8)		.22		0.6 (0.3-1.4)	
**Smoking**											
	Never smoked	35 (36.1)		23 (29.9)		Reference		—		Reference	
	Current or ex-smoker	62 (63.9)		54 (70.1)		0.9 (0.5-4.5)		.06		2.0 (0.9-4.3)	
**MLCCP^e^** **study sites**											
	WITS-CORE^f^	9 (9.3)		10 (13)		Reference		—		Reference	
	BMC^g^	88 (90.7)		67 (87)		2.8 (1.3-6.3)		.01		3.5 (1.3-9.3)^h^	
**Clinical staging**											
	I-II	3 (3.6)		2 (2.9)		Reference		—		Reference	
	III-IV	81 (96.4)		67 (97.1)		1.1 (0.3-4.5)		.62		1.6 (0.2-11.2)	
**ECOG^i^** **performance status**											
	0-2	60 (61.9)		63 (81.8)		Reference		—		Reference	
	3-4	37 (38.1)		14 (18.2)		2.8 (1.4-6.7)		.01		3.4 (1.2-6.6)^h^	
**Comorbidity**											
	No	44 (45.4)		35 (45.5)		Reference		—		Reference	
	Yes	53 (55.6)		42 (54.5)		1.3 (0.7-2.2)		.94		0.9 (0.5-1.9)	

^a^OR: odds ratio.

^b^aOR: adjusted odds ratio.

^c^Adjusted for age group, gender, education, occupation, smoking, Multinational Lung Cancer Control Program study site, clinical staging, Eastern Cooperative Oncological Group performance status, and comorbidity.

^d^Not applicable.

^e^MLCCP: Multinational Lung Cancer Control Program.

^f^WITS-CORE: University of the Witwatersrand Centre of Respiratory Excellence.

^g^BMC: Bugando Medical Centre.

^h^Statistically significant.

^i^ECOG: Eastern Cooperative Oncological Group.

### Association Between Symptom Burden and QoL

Symptom scores were significantly higher among patients with below-average QoL (<50) for fatigue, pain, dyspnea, insomnia, and appetite loss when compared with above-average QoL (≥50; Table 5). Higher symptom scores were associated with poor QoL.

**Table 5:** Associations between symptom burden and quality of life (QoL) of patients with lung cancer at 2 teaching hospitals across sub-Saharan Africa.

	Below-average QoL (<50; n=97), median (IQR)	Above-average QoL (≥50; n=77), median (IQR)	*P* value
Fatigue	66.67 (44.44-66.67)	44.44 (33.33-44.44)	<.001
Nausea and vomiting	0.00 (0.00-0.00)	0.00 (0.00-0.00)	.01^a^
Pain	66.67 (50.00-100.00)	50.00 (33.33-50.00)	<.001
Dyspnea	66.67 (33.33-66.67)	33.33 (0.00-33.33)	.004
Insomnia	66.67 (33.33-66.67)	33.33 (0.00-33.33)	<.001
Appetite loss	33.33 (0.00-66.67)	33.33 (0.00-33.33)	.001^a^
Constipation	0.00 (0.00-0.00)	0.00 (0.00-0.00)	.17
Diarrhea	0.00 (0.00-0.00)	0.00 (0.00-0.00)	.05
Financial difficulties	66.67 (33.33-66.67)	66.67 (33.33-66.67)	.01^a^

^a^Despite identical medians and IQRs in both groups (for nausea and vomiting, appetite loss, and financial difficulties), the statistical test revealed a significant difference (*P*<.005) due to the presence of small but systematic differences in the distribution of values—particularly in the frequency or magnitude of nonzero observations—that are not captured by median or IQR.

## Discussion

### Principal Findings

This study provides important insights into the QoL, symptom burden, and associated factors among patients with lung cancer across 2 sites in SSA. Overall, the findings demonstrated that QoL among participants was markedly low (median 41.67, IQR 33.33-41.67), substantially below the EORTC reference population, and varied between BMC and WITS-CORE [21]. Consistent with existing literature, a higher symptom burden, particularly pain, fatigue, dyspnea, insomnia, and appetite loss, was strongly associated with poorer QoL. Significant intersite differences were also observed in sociodemographic characteristics, clinical profiles, functional status, symptom experience, and financial challenges.

Regarding QoL domains, physical and role functioning were notably impaired, reflecting patients’ reduced capacity to perform daily activities, maintain independence, and fulfill social and family responsibilities. These findings are consistent with prior studies indicating that disease progression significantly compromises functional status. As lung cancer advances, patients often require assistance with basic activities, such as mobility, personal hygiene, and household responsibilities, which in turn affects their social roles and overall well-being [22]. Interestingly, cognitive functioning in this study appeared relatively preserved despite the high symptom burden. This aligns with some previous studies suggesting that cognitive domains may remain intact compared with physical and emotional domains [11,23-26]. However, this finding contrasts with evidence indicating that symptoms such as insomnia can negatively affect cognitive performance, while better cognitive functioning may help patients cope more effectively with stress and symptom distress [27]. This discrepancy highlights the need for more nuanced and longitudinal assessments of cognitive outcomes in this population.

The comparison between the 2 sites revealed both similarities and differences. Participants at both BMC and WITS-CORE were predominantly male, unemployed, and commonly presented without active tuberculosis, with many diagnosed at earlier stages (stage I-II). However, WITS-CORE participants generally had higher levels of education, were more likely to be smokers, and had a higher prevalence of comorbidities. These differences may reflect broader contextual factors, such as urban-rural disparities, health care accessibility, and diagnostic capacity. For example, the urban setting of WITS-CORE may facilitate earlier detection and access to specialized services, including advanced imaging and oncology expertise. In contrast, BMC serves a larger rural population, where geographical barriers, limited resources, and delayed health-seeking behaviors may influence disease presentation and outcomes. Previous studies have highlighted distance to health care facilities as a major barrier to timely cancer diagnosis and care in SSA [28].

Among sociodemographic factors, education level emerged as a significant determinant of QoL, while other variables, such as age, sex, and economic status, showed inconsistent associations, reflecting mixed findings in the literature [11,21,29,30]. Higher education may enhance health literacy, enabling patients to better understand their condition, seek timely care, adhere to treatment, and engage in self-management [31]. The lower education levels observed among BMC participants, who largely came from rural communities, underscore the need for targeted health education interventions delivered in local languages to improve awareness, early detection, and ultimately QoL outcomes.

Symptom burden in this study was substantial, particularly among patients with advanced disease, and aligned with global evidence identifying fatigue, pain, dyspnea, insomnia, and appetite loss as the most prevalent and distressing symptoms in lung cancer [7,11,22,32,33]. Notably, pain and insomnia were more severe among patients at BMC, possibly reflecting disparities in access to pain management and supportive care services. In many settings in SSA, effective pain control remains a challenge due to regulatory restrictions on opioid availability, limited health care provider training, and financial constraints faced by patients [34]. Addressing these barriers through policy reforms, task sharing in opioid prescribing, and capacity building among health care providers is essential. Given the strong association between symptom burden and poor QoL observed in this study, integrating palliative care into routine cancer services is critical to ensure effective symptom management and improve patient outcomes [35-37].

Financial hardship was widely reported, particularly among BMC participants, and is consistent with the high unemployment rate observed in the study population. Although financial difficulties were not statistically associated with QoL in this analysis, extensive evidence indicates that economic burden significantly affects access to care, treatment adherence, and overall well-being [21,25,34,38,39]. Financial challenges may include direct health care costs as well as indirect costs, such as transportation, nutrition, and loss of income. Differences in social protection systems, such as disability grants available in South Africa, may partly explain variations between sites. Expanding financial support mechanisms for patients with life-limiting illnesses across SSA could play a crucial role in improving QoL.

### Limitations

This study has several limitations. The relatively small sample size at BMC may limit generalizability and statistical power. Although a standardized tool (EORTC QLQ-C30) was used, differences in interview languages across sites may have influenced data consistency. Variability in diagnostic and staging capabilities between facilities may also affect the accuracy of clinical classification. Additionally, some relevant factors such as cough, treatment-related effects, nutritional status, use of alternative therapies, and polypharmacy were not assessed but may influence QoL. The cross-sectional design further limits the ability to establish causal relationships or assess changes over time. Future prospective or longitudinal studies are therefore recommended to better understand causal pathways and temporal dynamics of QoL and symptom burden. Despite these limitations, this study represents one of the first multicountry analyses of QoL and symptom burden among patients with lung cancer in SSA. The findings underscore the urgent need for integrated, patient-centered cancer care that prioritizes symptom management, strengthens health system capacity, addresses socioeconomic barriers, and incorporates palliative care as a core component of oncology services.

### Conclusions

In consistency with previous studies, this study revealed disparities in QoL and symptom burden among patients with lung cancer in SSA. Overall, patients experienced low QoL, functional impairment, and financial impact, with prevalent symptoms such as pain, fatigue, dyspnea, insomnia, and appetite loss. Variations in education levels, health care access, and site-specific factors further underscored inequalities in cancer care across the region. These findings highlight the pressing need for context-sensitive interventions that strengthen palliative care services, enhance cancer health literacy through community-based education, and expand social and financial support for patients. Collectively, the study offers valuable regional evidence to guide policies and practices aimed at improving the QoL and clinical outcomes of patients with lung cancer in SSA.

## Abbreviations

aOR: adjusted odds ratio
BMC: Bugando Medical Centre
CORE: Centre of Respiratory Excellence
ECOG-PS: Eastern Cooperative Oncological Group Performance Scale
EORTC QLQ-C30: European Organization for Research and Treatment of Cancer Quality of Life Questionnaire Core 30
MLCCP: Multinational Lung Cancer Control Program
NSCLC: non–small cell lung cancer
OR: odds ratio
QoL: quality of life
SSA: sub-Saharan Africa
WITS: University of the Witwatersrand

## Data Availability

The datasets used and/or analyzed during this study are available from the corresponding author on reasonable request.
